# Differential modulation of host immune genes in the kidney and cranium of the rainbow trout (*Oncorhynchus mykiss*) in response to *Tetracapsuloides bryosalmonae* and *Myxobolus cerebralis* co-infections

**DOI:** 10.1186/s13071-018-2912-7

**Published:** 2018-05-30

**Authors:** Mohamed H. Kotob, Gokhlesh Kumar, Mona Saleh, Bartolomeo Gorgoglione, Mahmoud Abdelzaher, Mansour El-Matbouli

**Affiliations:** 1 Clinical Division of Fish Medicine, University of Veterinary Medicine, Veterinärplatz 1, 1210 Vienna, Austria; 2 Department of Pathology, Faculty of Veterinary Medicine, Assiut University, Assiut, 71526 Egypt; 3 Department of Biological Sciences, Wright State University, Dayton, OH 45435 USA

**Keywords:** Co-infections, Salmonids, Proliferative kidney disease, Whirling disease, JAK/STAT signaling pathway, Immunosuppression

## Abstract

**Background:**

Most of the studies on fish diseases focus on single infections, although in nature co-infections occur more often. The two freshwater myxozoan parasites of salmonids, having high economic and ecologic relevance are *Tetracapsuloides bryosalmonae* (Malacosporea), the etiological agent of proliferative kidney disease, and *Myxobolus cerebralis* (Myxosporea), the etiological agent of whirling disease. The present study aims to investigate immune modulation in rainbow trouts (*Oncorhynchus mykiss*) during single and co-infections by these parasites.

**Methods:**

Fish were initially infected with *T. bryosalmonae* (one group) and *M. cerebralis* (another group) separately*.* At 30 days post-exposure (dpe), both the single species infected groups were co-infected, respectively, with the other parasite. Posterior kidney and cartilage cranium samples were collected at 30, 60, 90 and 120 dpe and RT-qPCR was performed on them to assess the transcription of suppressors of cytokine signaling (SOCS) -1 and -3, Janus kinase-1 (JAK-1) and signal transducer and activator of transcription-3 (STAT-3) genes.

**Results:**

Kidney samples from the *T. bryosalmonae-*infected group showed upregulation of all immune genes tested between 60–120 dpe. Crania from the single *M. cerebralis-*infected group and the *M. cerebralis* and *T. bryosalmonae* co-infected group exhibited upregulation of SOCS-1 and JAK-1 between 60–120 dpe and SOCS-3 at 120 dpe. However, only in the single *M. cerebralis-*infected group, was a statistically significant expression of STAT-3 observed at 30 and 60 dpe.

**Conclusions:**

The results of this study indicate that both *T. bryosalmonae* and *M. cerebralis* induce overexpression of SOCS-1 and SOCS-3 genes and modulate the host immune response during the development of parasite to cause immunosuppression.

**Electronic supplementary material:**

The online version of this article (10.1186/s13071-018-2912-7) contains supplementary material, which is available to authorized users.

## Background

Proliferative kidney disease (PKD) is caused by the myxozoan parasite, *Tetracapsuloides bryosalmonae* that belongs to the class Malacosprea and phylum Myxozoa [1, 2]. PKD causes high economic losses of farmed and wild autochthonous salmonids and is distributed in the regions of Europe and North America [3–5]. The life-cycle of *T. bryosalmonae* alternates between an invertebrate freshwater bryozoan host (*Fredericella sultana*) and a vertebrate salmonid host [4, 6]. PKD targets the kidney and induces chronic immunopathology, granulomatous-like lesions and lymphocytic hyperplasia of the interstitial kidney tissue, along with hyperimmunoglobulinemia [7, 8]. Since this disease is temperature-dependent, climate change plays a crucial role in its pathogenesis [5]. The infective *T. bryosalmonae* malacospores spread either through infected tolerant hosts, such as brown trout (*Salmo trutta*) [9], or infected bryozoan dispersal, *via* migrating zooids [10] and infected statoblasts [11]. During PKD the mortality rate can range from less than 20%, to 95–100% in serious outbreaks that are complicated by secondary infections and unfavorable farming or environmental conditions [5, 12, 13].

Fish that recover from PKD acquire a strong immunity and become resistant to re-infections [14]. This results from the massive activation of B cells, which induces hyperimmunoglobulinemia in response to the parasite’s extra-sporogonic histozoic proliferation [8, 15, 16]. Rainbow trout (*Oncorhynchus mykiss*) show upregulation of tumor necrosis factor (TNF-α2), cyclooxygenase (COX-2) and, to some degree, of transforming growth factor (TGF)-β, upon natural infection with *T. bryosalmonae* [8, 17]. The quality of the immune reaction to *T. bryosalmonae* is temperature-dependent, resulting in either a predominant Th2-like immune response with abundant B cell response at 15 °C or predominant Th1-like immune response with upregulation of the natural killer cell enhancement factor (NKEF) at 12 °C [18].

Whirling disease (WD) is a highly debilitating disease of salmonids that is caused by the myxozoan parasite *Myxobolus cerebralis* [19, 20]. *M. cerebralis* alternates between two hosts, an invertebrate oligochaetae host (*Tubifex tubifex*) and a vertebrate salmonid host, to complete a complex life-cycle [21, 22]. Mature myxospores that are formed within the fish cartilage can infect *T. tubifex* and once inside, *T. tubifex* triactinomyxons (TAMs) are formed and released into the water, thus infecting salmonids [23, 24]. WD is implicated in the decline of wild trout populations in North America [22]. The severity of WD depends largely on the age and size of the affected fish, with higher mortality rates in fingerlings, up to 90% of the infected populations [22].

The binding of a wide array of cytokines and growth factors to their cell receptors activates the associated Janus kinase (JAK) proteins and subsequently phosphorylates the cytokine receptor complex. Thereafter, the binding of signal transducer and activator of transcription (STAT) proteins to the site of the activated receptor and their phosphorylation occurs. Activated STAT proteins translocate to cell nucleus for signal transduction and initiate the gene transcription [25–27]. The suppressors of cytokine signaling (SOCS) are a group of intracellular molecules that act as strong negative regulators of the cytokine signaling through inhibition of the JAK-STAT pathway [28]. Even in Teleost fish, SOCS molecules are known to play an important role during the development of innate and acquired immunity [29], with SOCS-1 and SOCS-3 being selectively induced during PKD [8, 30].

Even though co-infections occur frequently in the aquatic environment, the research on such a subject is still in the infancy, with most of the studies often being conducted on single infections [31–33]. During co-infections, the host immune response induced by one pathogen can alter the pathogenesis of the secondary infections through the suppression or stimulation of the immune system [31–33]. The interaction between different parasites could be either synergistic or antagonistic in the infected host [32, 33]. Mixed infection of five myxozoan parasite species: *T. bryosalmonae*, *Sphaerospora truttae*, *Chloromyxum schurovi*, *C. truttae,* and *Myxobolus* sp. has been examined in farmed brown trout (*Salmo trutta*) [34]. A big knowledge gap still exists in the understanding of immune response mechanism in salmonids, during co-infections. Consequently, a substantial need for investigating the interactions between heterogeneous micro-organisms during co-infections, is there [35].

Recently, the impact of *T. bryosalmonae* and *M. cerebralis* co-infection on pathology of the target organs of rainbow trout has been examined [36]. The present study was designed to examine the expression of immune genes in rainbow trout when co-infected with two myxozoan parasites, by measuring the transcription levels of JAK/STAT signaling induced genes and SOCS genes in the posterior kidney and cranial cartilages (the target tissues of PKD or WD pathogenesis), respectively.

## Methods

### Fish and the experiment design

**Fig. 1:**
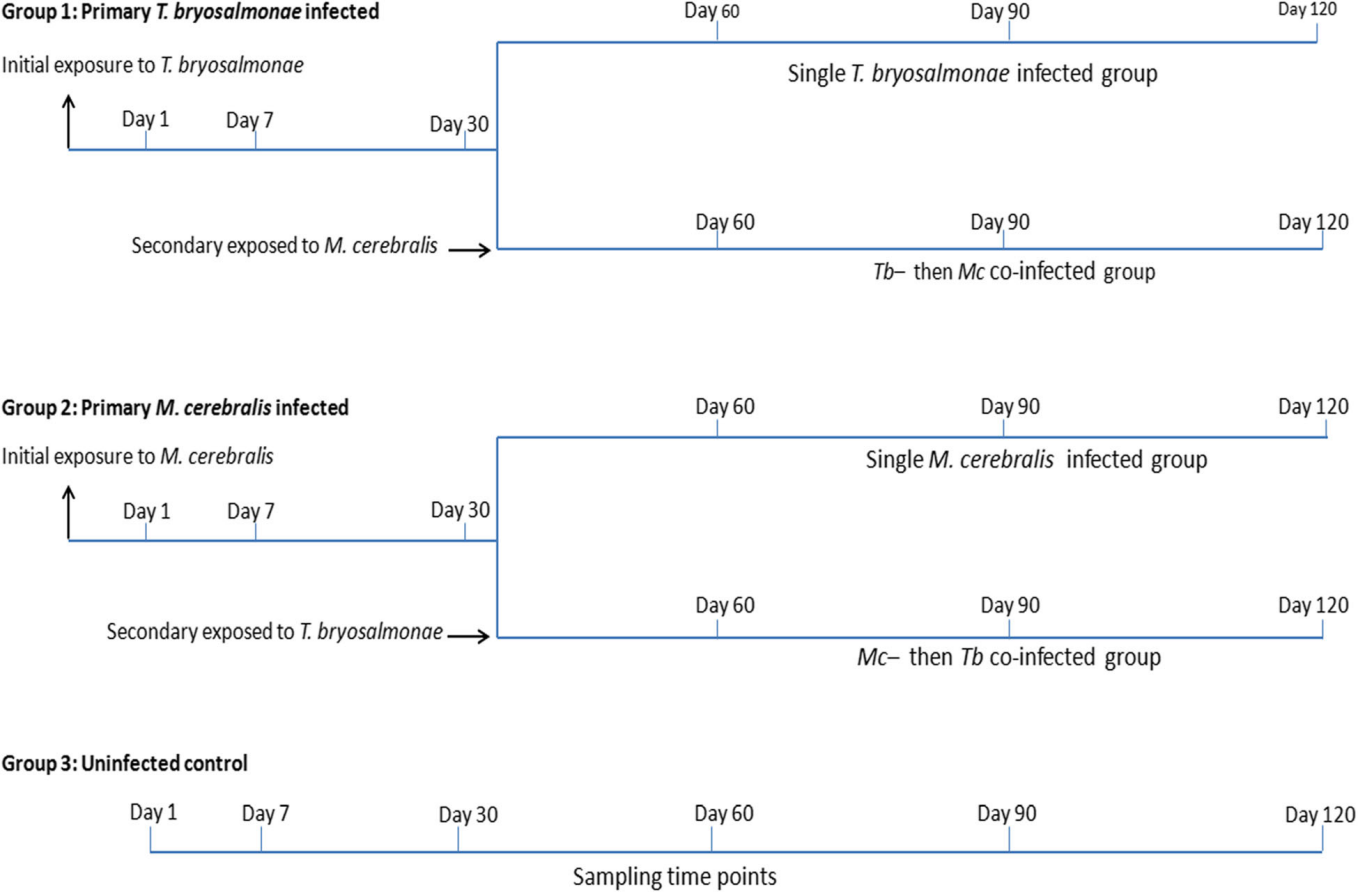
The experimental design. Three primary groups of rainbow trout: *T. bryosalmonae-*infected, *M. cerebralis-*infected, and uninfected control. Primary infected fish were exposed to *M. cerebralis* and *T. bryosalmonae* at 30 dpe, thereafter fish were sampled at the specified time points (adapted from Kotob et al. [36])

The experimental challenges, consisting of single infection and co-infection with parasites, were performed as described previously in the first part of this study and the focus was on the pathological assessment (Fig. 1) [36]. Briefly, pathogen-free rainbow trout (mean length 4.02 ± 0.26 cm, mean weight 0.6 ± 0.15 g) were divided into three groups of 96 each: the first group was infected with *T. bryosalmonae* spores, according to Kumar et al. [37], while the second group was infected with *M. cerebralis* TAMs, according to Hedrick et al. [38], and the third group was kept as uninfected control*.* Thirty days later, half of the fish from the first two infected groups were reciprocally co-infected with these parasites. At 30, 60, 90 and 120 days post-exposure (dpe), the fish were euthanized using an overdose of tricaine methanesulfonate (500 mg/l, MS-222, Sigma-Aldrich, Steinheim, Germany) and posterior kidneys and crania were dissected out and preserved in RNAlater (Sigma-Aldrich).

### RNA extraction and cDNA synthesis

Total RNA was extracted from the posterior kidneys and cranial samples of each group (*n* = 4) at each time point, starting from 30 dpe (time of co-infection), using RNeasy Mini Kit (Qiagen, Hilden, Germany) following the manufacturer’s instructions. An on-column DNase digestion step was also included in order to remove any residual DNA contamination. RNA concentration was determined using a Nanodrop 2000c spectrophotometer (Thermo Fisher Scientific, Wilmington, USA) and one microgram of total RNA was used to synthesize cDNA, with iScript cDNA Synthesis Kit (Bio-Rad, Hercules, USA).

### Reverse transcription quantitative PCR (RT-qPCR)

Parasite load determination (respectively for *T. bryosalmonae* and *M. cerebralis*) and quantification of the expression of SOCS-1, SOCS-3, JAK-1 and STAT-3 genes, were performed using samples from posterior kidneys and the crania of infected fish, following the previously described methodology [8, 39–41]. Primers used for the gene transcription assessment are summarized in Table 1. The quantity of gene expression level was measured with CFX96 Touch Real-Time PCR detection system (Bio-Rad). The PCR reaction of 20 μl final volume contained 4 μl of 1:10-fold diluted cDNA, 1× SsoAdvanced Universal SYBR Green Supermix (Bio-Rad), 0.4 μM of each primer, and DEPC-treated sterile distilled water (Bio-Rad). The PCR reaction consisted of an initial 5 min of cDNA denaturation at 95 °C, followed by 35 cycles of 95 °C for 30 s, 57–62 °C for 30 s and 72 °C for 30 s. A melting-point curve was measured, starting from 57 °C with an increase of 0.5 °C at every 10 s up to 95 °C, for detecting non-specific binding. Elongation factor 1 alpha [42] was used as a reference gene for normalizing the expression of targeted genes and calculation of relative gene expression was done using CFX manager software version 3.1 (Bio-Rad).

**Table 1:** List of quantitative real-time PCR primers

Primer name	Primer sequence (5′-3′)	Amplicon size (bp)	GenBank ID	Reference
*T. bryosalmonae* RPL18 F	GTAAACGGGGACAAAAAGA	251	FR852769	[8]
*T. bryosalmonae* RPL18 R	GGAGCAGCACCAAAATAC			
Myx18-909 F	CTTTGACTGAATGTTATTCAGTTACAGCA	88	AF115253	[39]
Myx18-996 R	GCGGTCTGGGCAAATGC			
SOCS-1 F	GATTAATACCGCTGGGATTCTGTG	136	AM748721	[8]
SOCS-1 R	CTCTCCCATCGCTACACAGTTCC			
SOCS-3 F	CACAGAGAAACCGTTAAAAGGACTATCC	228	AM748723	[8]
SOCS-3 R	AAGGGGCTGCTGCTCATGAC			
JAK-1 F	ACACTGATATTGGGCCGTTCTGGA	174	CA378782	[40]
JAK-1 R	CCTCGTCCTCTGCATCTTTACCAAC			
STAT-3 F	GAATGAAGGGTATATTCTGG	152	U60333	[41]
STAT-3 R	TCCCACTGATGTCCTTTTCC			
EF-1α F	AGACAGCAAAAACGACCCCC	167	HF563594	[42]
EF-1α R	AACGACGGTCGATCTTCTCC			

These primers were used in RT-qPCR to quantify the relative gene expression in posterior kidneys and cranial cartilages of infected rainbow trout during single and co-infections

### Statistical analysis

The differences in the expression of genes that were tested at different time points for each group were analyzed by a general linear model with repeated measurements and Sidak’s procedure was used for multiple comparison. One-way ANOVA with Tukey’s α-correction was used to examine the differences of expressions between all groups at each time point. Pearson’s product-moment correlation coefficient (*r*) was measured to determine the correlation between relative expressions of all tested immune genes. The statistical differences were considered significant at a *P*-value < 0.05 and all the data were analyzed in IBM SPSS software version 24.

## Results

### *Tetracapsuloides bryosalmonae* and *Myxobolus cerebralis* parasite burden

**Fig. 2:**
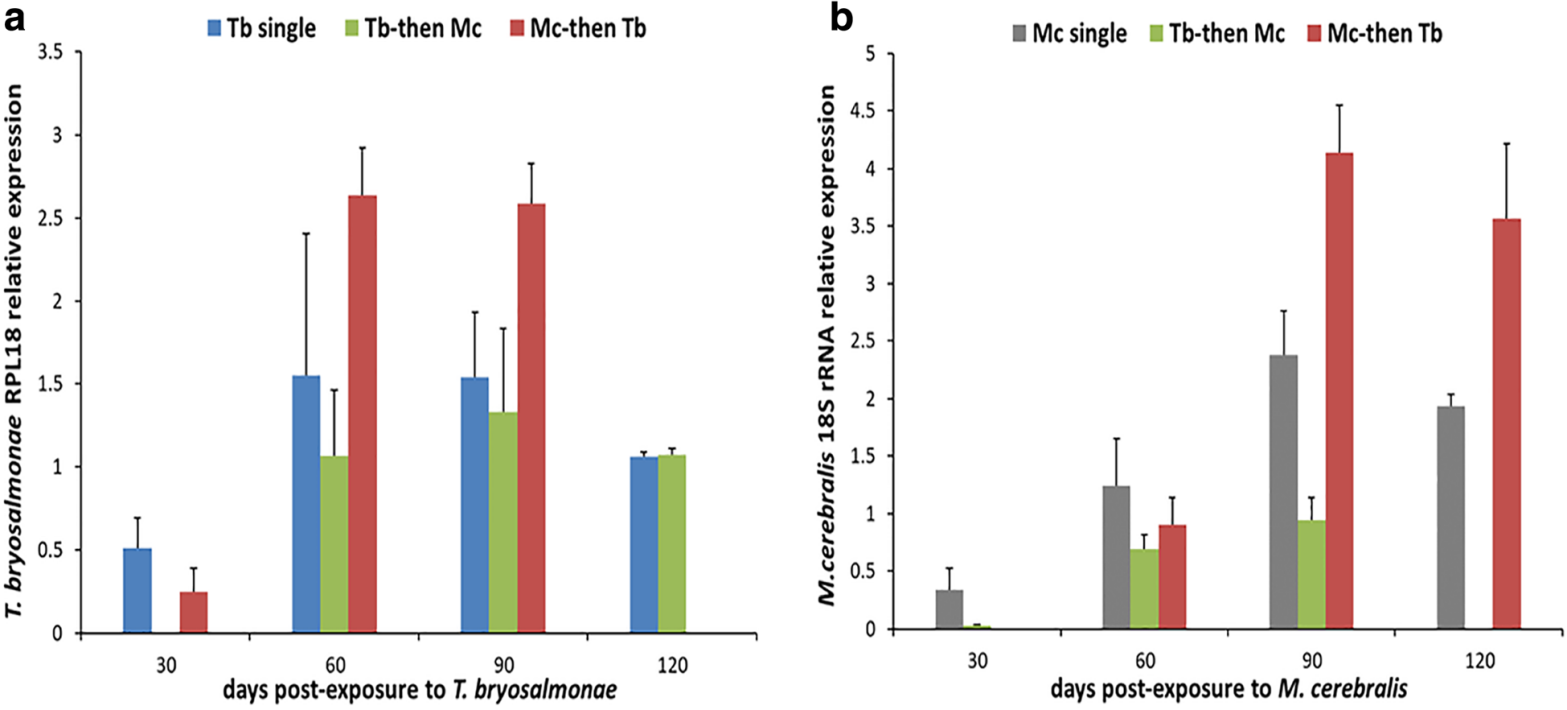
Parasite burden quantification in the posterior kidneys and cranial cartilages of infected rainbow trout. **a** *T. bryosalmonae* RPL18 expression in posterior kidneys of *T. bryosalmonae-*infected rainbow trout, **b** *M. cerebralis 18S* rRNA expression in cranial cartilages of *M. cerebralis-*infected rainbow trout. Bars indicate standard deviation (*n* = 4)

The relative expression of 60S ribosomal protein L18 of *T. bryosalmonae* (RPL18) and *18S* rRNA genes of *M. cerebralis* in the posterior kidneys and the crania, respectively, showed an increasing burden during the pathogenesis progression in single and co-infections (Fig. 2). *Tb* burden reached significantly higher levels in the *Mc-*then-*Tb* co-infected group, in the posterior kidneys during 60 and 90 days post secondary exposure to *T. bryosalmonae* (*F*_(2, 6)_ = 5.969, *P* < 0.05; *F*_(2, 6)_ = 8.750, *P* < 0.05) (Fig. 2a). *Mc* burden in the *Tb*-then-*Mc* co-infected group was significantly lower than the single *Mc-*infected group at 30 dpe to *Mc* (*F*_(1, 4)_ = 8.240, *P* = 0.045), then *Mc* burden increased at 60 dpe to *Mc* in all *Mc-*infected groups and reached significantly higher levels in *Mc-*then-*Tb* co-infected group at 90 and 120 dpe to *Mc* (*F*_(2, 6)_ = 64.105, *P* < 0.01; *F*_(1, 4)_ = 18.489, *P* = 0.013, respectively) (Fig. 2b).

### Immune gene expression in the posterior kidneys during single and co-infections

**Fig. 3:**
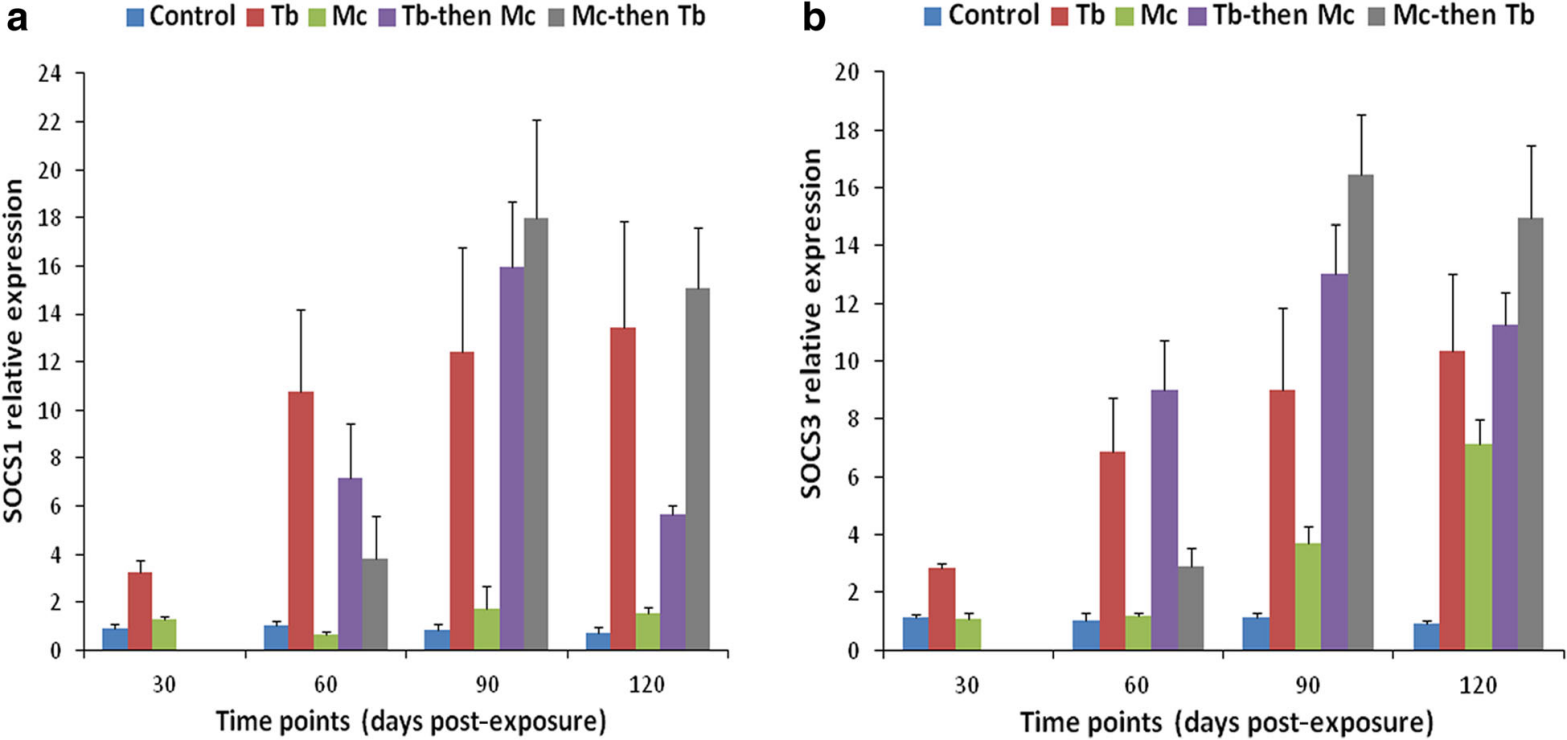
Relative gene expression of SOCS-1 (**a**) and SOCS-3 (**b**) in posterior kidneys during single and co-infections. RT-qPCR data from different time points were normalized to EF-1α expression and relative gene expression data were statistically analyzed. Bars indicate standard deviation (*n* = 4). Time points were calculated from the primary exposures to *T. bryosalmonae* and *M. cerebralis*

**Fig. 4:**
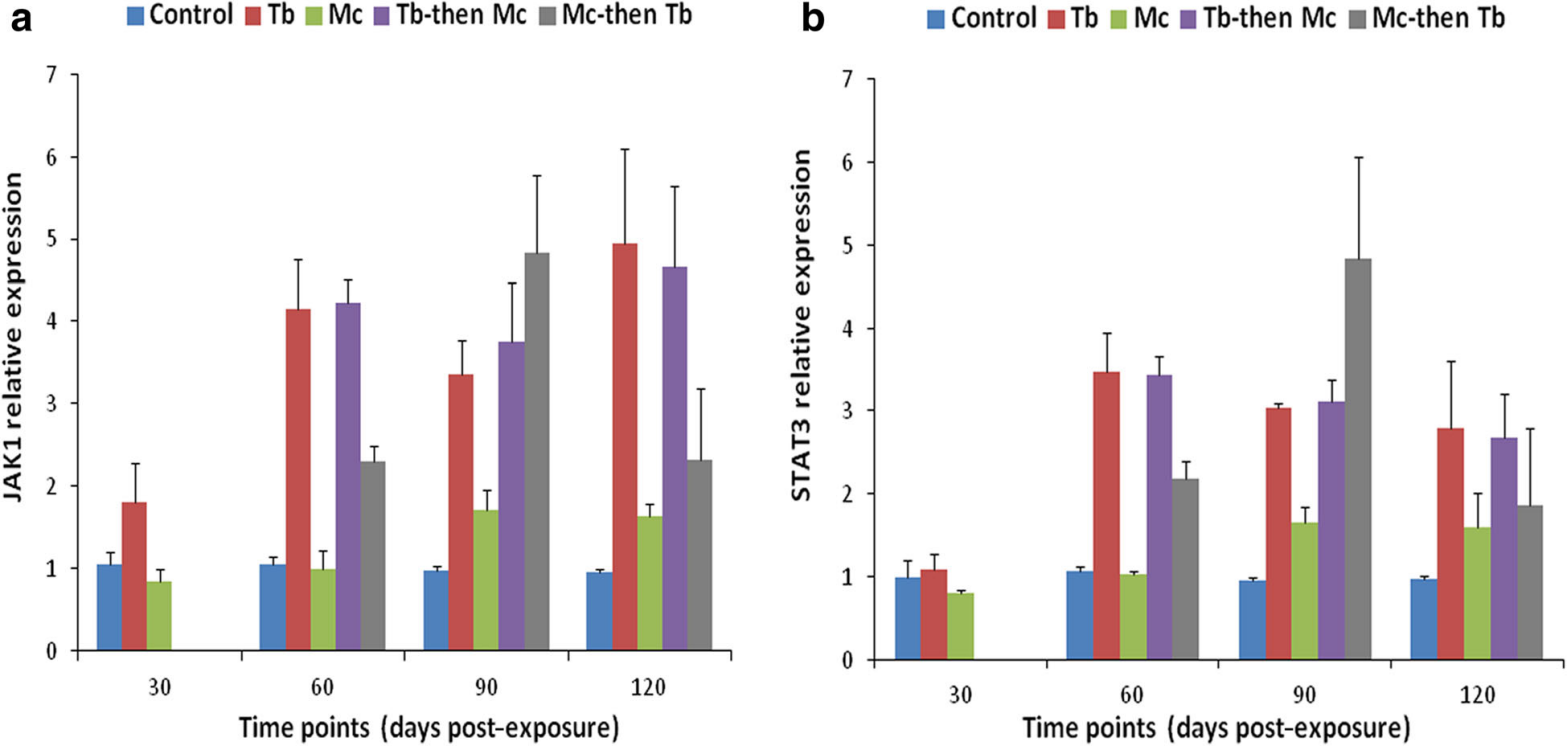
Relative gene expression of JAK-1 (**a**) and STAT-3 (**b**) in posterior kidneys during single and co-infections. Details are the same as in Fig. 3

Kidneys of the single *Mc*-infected group displayed no significant differences in the expression of SOCS-1, JAK-1 and STAT-3 genes at all the time points, when compared to the uninfected control (*F*_(1, 4)_ = 2.670, *P* > 0.05). However, only SOCS-3 gene expression increased from 90 dpe and showed a significant increase at 120 dpe (*F*_(1, 4)_ = 158.623, *P* = 0.001), indicating the stimulation of systemic immune response during the late stages of WD (Figs. 3, 4).

The expression of SOCS-1, SOCS-3 and JAK-1 genes started to increase in the single *Tb-*infected group compared to the single *Mc-*infected and uninfected control groups at 30 dpe (*F*_(2, 6)_ = 46.157, *P* < 0.001 for SOCS-1; *F*_(2, 6)_ = 108.095, *P* < 0.001 for SOCS-3 and *F*_(2, 6)_ = 8.116, *P* < 0.05 for JAK-1). No significant difference in the transcription of STAT-3 gene was observed between all the groups at this stage (*F*_(2,6)_ = 2.471, *P* = 0.165) (Figs. 3, 4).

At 60 dpe, SOCS-1 gene expression was upregulated in all *Tb-*infected groups which was significant in the single *Tb-*infected group compared to single *Mc-*infected and uninfected control groups (*F*_(2, 6)_ = 25.766, *P* = 0.001), and other co-infected groups (*F*_(2, 6)_ = 5.486, *P* = 0.044). When all the groups were compared, the upregulation of SOCS-3 was significant in the single *Tb* and *Tb*-then-*Mc* co-infected groups (*F*_(4, 10)_ = 27.941, *P* < 0.01) and upregulation of JAK-1 and STAT-3 gene expression was significant in all *Tb-*infected groups (*F*_(4, 10)_ = 56.291, *P* < 0.01 for JAK-1 and *F*_(4, 10)_ = 58.690, *P* < 0.01 for STAT-3) (Figs. 3, 4).

At 90 dpe, increased gene expression of SOCS-1, SOCS-3, JAK-1 and STAT-3 transcriptions was seen in all the *Tb-*infected groups, especially in the *Mc*-then-*Tb* co-infected group, compared to the uninfected control and single *Mc*-infected groups (*F*_(4, 10)_ = 21.612, *P* < 0.001 for SOCS-1; *F*_(4, 10)_ = 38.323, *P* < 0.001 for SOCS-3; *F*_(4, 10)_ = 22.507, *P* < 0.001 for JAK-1 and *F*_(4, 10)_ = 20.744, *P* < 0.001 for STAT-3) (Figs. 3, 4).

At 120 dpe, all the *Tb-*infected groups showed significant upregulation of SOCS-1 and SOCS-3 gene expression when compared with the uninfected control group (*F*_(4, 10)_ = 24.981, *P* < 0.05 for SOCS-1 and *F*_(4, 10)_ = 27.407, *P* < 0.01 for SOCS-3). Moreover, gene expression of JAK-1 and STAT-3 genes were upregulated in the single *Tb-*infected and *Tb*-then-*Mc* co-infected groups (*F*_(4, 10)_ = 15.852, *P* < 0.01 for JAK-1 and *F*_(4, 10)_ = 4.417 *P* < 0.05 for STAT-3) compared to other groups (Figs. 3, 4).

The correlation of tested immune genes was positively strong between SOCS-1 and JAK-1 in the single *Tb-*infected group (*r* = 0.619, *P* = 0.032) (see Additional file 1: Table S1). The correlation between the expression of SOCS-1 and STAT-3 genes was also highly positive in the single *Tb-*infected group (*r* = 0.577 and *P* = 0.049). Interestingly, a strong and highly significant positive correlation was detected in expression of SOCS-1 and SOCS-3 genes in the single *Tb-*infected and *Mc-*then-*Tb* co-infected groups (*r* = 0.959 and *P* = 0.0001; *r* = 0.895 and *P* = 0.001, respectively). SOCS-3 correlated positively with JAK-1 and this correlation was highly significant in the single *Mc-*infected group (*r* = 0.785 and *P* = 0.002). A weak positive correlation was found between SOCS-3 and STAT-3 in all groups except in the single *Mc-*infected group, where the correlation was strong (*r* = 0.722 and *P* = 0.008) and the expression of both genes was found to be high at 90 and 120 dpe. Strong positive correlation was also found between JAK-1 and STAT-3 in single *Tb* and *Mc-*infected groups (*r* = 0.779 and *P* = 0.003; *r* = 0.775 and *P* = 0.003, respectively) (see Additional file 1).

### Immune gene expression in the crania during single and co-infections

**Fig. 5:**
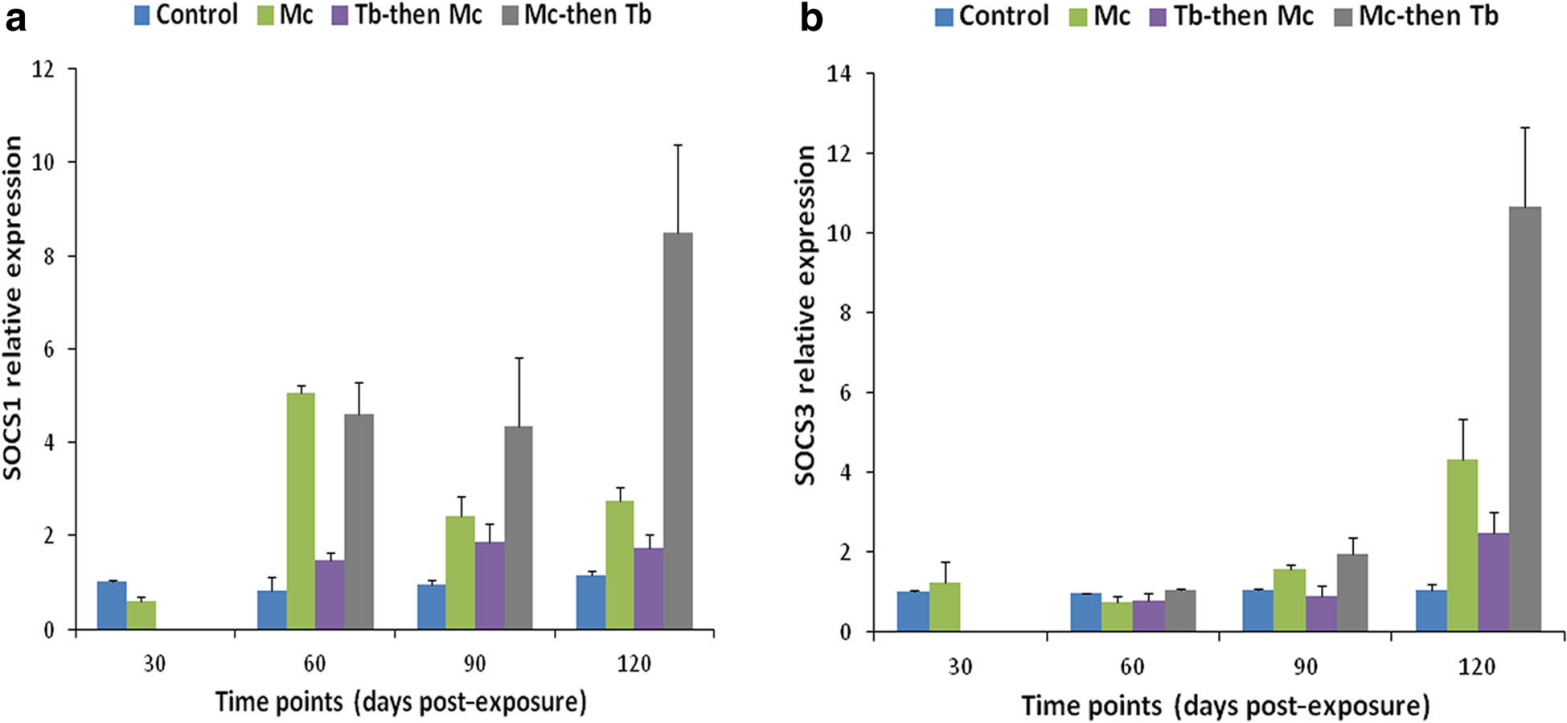
Relative gene expression of SOCS-1 (**a**) and SOCS-3 (**b**) in cranial cartilages during single and co-infections. Details are the same as in Fig. 3

The transcription of SOCS-1 and SOCS-3 genes was significantly higher between 60–120 dpe (*F*_(3, 8)_ = 92.682, *P* < 0.001 at 60 dpe; *F*_(3, 8)_ = 9.596, *P* < 0.01 at 90 dpe and *F*_(3, 8)_ = 36.842, *P* < 0.001 at 120 dpe) (Fig. 5a) and at 120 dpe in the single *Mc* and *Mc*-then-*Tb* co-infected groups (*F*_(3, 8)_ = 39.441, *P* < 0.01) (Fig. 5b), respectively.

**Fig. 6:**
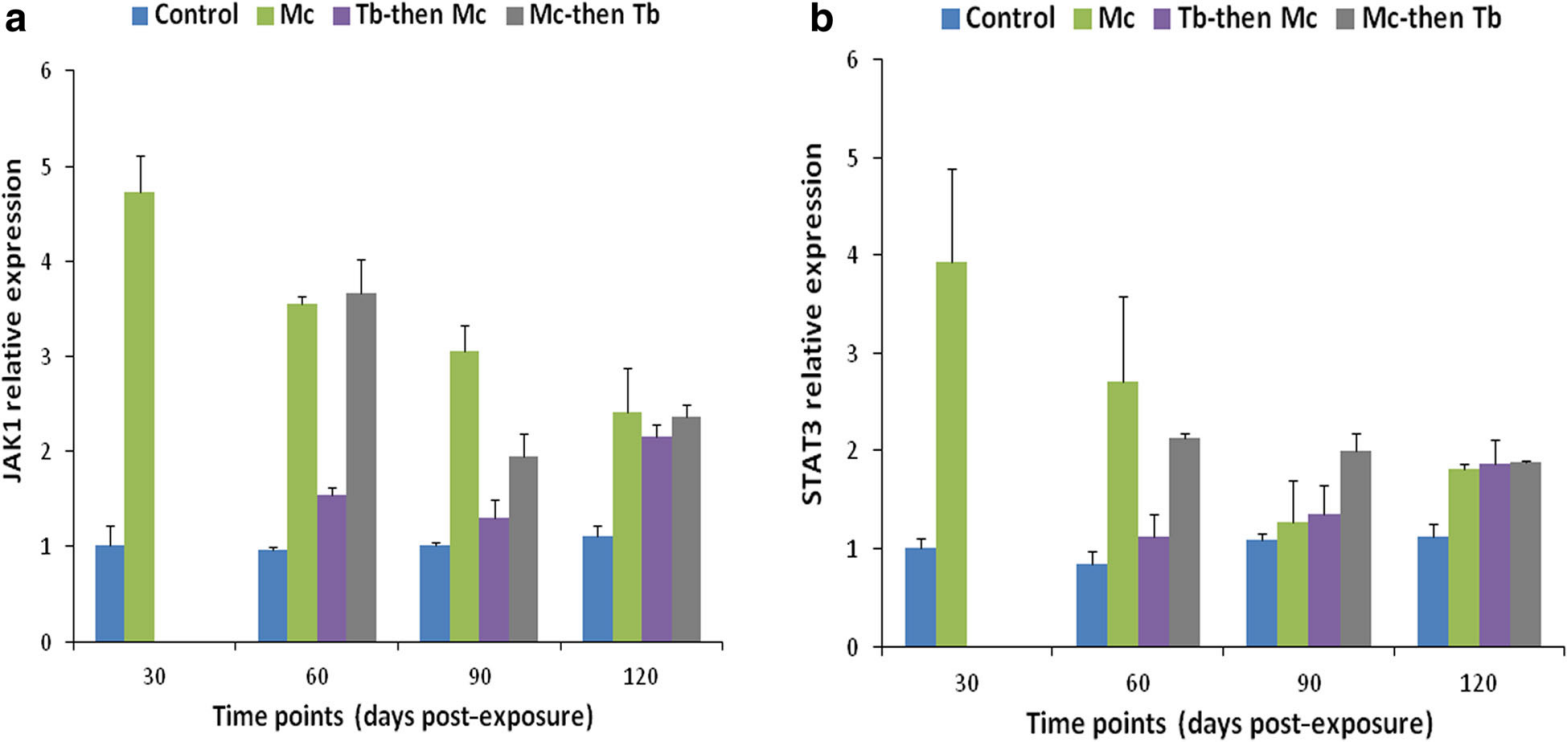
Relative gene expression of JAK-1 (**a**) and STAT-3 (**b**) in cranial cartilages during single and co-infections. Details are the same as in Fig. 3

In the case of JAK-1 and STAT-3 gene expression, a remarkable increase was observed in the single *Mc-*infected group at 30 dpe (*F*_(1, 4)_ = 713.629, *P* < 0.001 for JAK-1 and *F*_(1, 4)_ = 1489.910, *P* < 0.0001). The transcription of JAK-1 gene was significantly increased between 60–120 dpe in the single *Mc* and *Mc*-then-*Tb* co-infected groups compared to other groups (*F*_(3, 8)_ = 36.765, *P* < 0.001 at 60 dpe; *F*_(3, 8)_ = 54.622, *P* < 0.001 at 90 dpe and *F*_(3, 8)_ = 16.834, *P* < 0.01 at 120 dpe) (Fig. 6a). However, statistically significant expression of STAT-3 was only observed in the single *Mc-*infected group which continued at 60 dpe (*F*_(3, 8)_ = 29.919, *P* < 0.001) (Fig. 6b).

Since there was a low level of expression of all immune genes in the cranial cartilages, we did not observe any correlation between the immune genes expression.

## Discussion

Myxozoan parasites can modulate the pro-inflammatory cellular responses including phagocytosis, oxidative phagocytic activity and complement activity [43]. Additionally, lysozyme, peroxidases, and acute-phase proteins, as the players of humoral immune mechanisms, are also involved in the immune response against those parasites [8, 43]. In this study, we have investigated, for the first time, the immune response of rainbow trout during single and co-infections between *T. bryosalmonae* and *M. cerebralis,* focusing on selective genes that are involved in the SOCS/JAK/STAT signaling pathway. We also determined the correlations of gene expression between these immune-related genes. The data obtained from this study demonstrate that the posterior kidneys of all *T. bryosalmonae-*infected fish had elevated levels of tested immune genes and that the pathway was highly induced during the pathogenesis of PKD, which was present either as a single disease or in concomitance with WD. However, the relative gene expressions of SOCS-1 and SOCS-3 were much higher than JAK-1 and STAT-3. Additionally, the highest load of both parasites was detected in fish co-infected initially with *M. cerebralis* and then with *T. bryosalmonae* at 90 dpe. The kidneys of fish from this group showed increased expressions of tested immune genes at 90 dpe and their crania also showed the highest gene expression levels of SOCS-1 and SOCS-3 at 120 dpe.

The transcription level of the *T. bryosalmonae* RPL18 gene in cDNAs was measured to assess the relative burden of the parasite *T. bryosalmonae* in posterior kidneys [8]. The single *Tb-*infected and *Tb*-then-*Mc* co-infected groups showed a decrease in the gene expression of *T. bryosalmonae* RPL18 at 90 and 120 dpe, suggesting the activation of immune system and parasite clearance during this time. However, this was not in agreement with the immunohistochemistry (IHC) results obtained by counting the number of parasites [36]. The disparity between both the results could be because of the fact that the IHC data reflected both dead and viable parasites while the data obtained by *T. bryosalmonae* RPL18 gene expression reflected only viable parasites in the samples. Nevertheless, the relative gene expression of *T. bryosalmonae* RPL18 in *Mc*-then-*Tb* co-infected group persisted to increase, indicating active parasite proliferation and the suppression of immune response. Furthermore, the primary infection of rainbow trout with *M. cerebralis* and then with *T. bryosalmonae* fostered the pathogenesis of both infections and this co-infected group exhibited high parasitic burden of both parasites (Fig. 2a, b). These data were in accordance with the counting of *T. bryosalomae* stages in the kidney of infected fish [36]. The synergistic interaction that occurred in this case of co-infection might be due to the immunosuppressive effect of secondary exposure to *T. bryosalmonae,* which was 30 days after primary *M. cerebralis* exposure [8, 17, 44]. PKD-mediated immunosuppression occurs due to the downregulation of some key regulatory genes and significant decrease in phagocytic and respiratory burst activity of kidney macrophages [44]. During the course of primary infection with *M. cerebralis*, the spores successfully parasitize and multiply in the cranium and evade the immune system before the secondary exposure to *T. bryosalomonae*, because of which the infected fish exhibits exacerbated form of clinical signs and lesions of both diseases along with the highest levels of transcription of the specific genes of parasites load (*T. byrosalmonae* RPL18 and *M. cerebralis 18S* rRNA) [36]. On the contrary, the secondary infection with *M. cerebralis* counteracts the development and the load of *M. cerebralis* in the cranium of *Tb-*infected rainbow trout, evident from the lower parasitic load in this group (Fig. 2b). The decreased parasitic burden could be due to the cross-reactivity between the sporogonic stages of both parasites that might induce cross-immunity and thereby interfering with the pathogenesis and parasitic burden of secondary *M. cerebralis* [45].

The pathogenesis of PKD is characterized by an anti-inflammatory reaction, T helper cell-like activity and an intense B cell/antibody response with a marked upregulation of interleukin (IL)-6, IL-10, IL-11 and antimicrobial peptides [8]. In our study, we found that the expression of SOCS-1 and SOCS-3 genes was higher during the progression of PKD pathogenesis, further reaffirming the fact that the parasite causes an immunosuppression reaction in host in order to evade its immune system. This agrees with previous studies that have demonstrated specifically that the expression of SOCS-1 and SOCS-3 genes strongly correlate with the parasitic burden and pathology progression [8, 30]. In addition, one of the most striking findings was the presence of increased SOCS-1 and SOCS-3 gene transcription in the *Mc*-then-*Tb* co-infected group at 90 dpe that correlated positively with the kidney swelling index and pathological lesions of kidneys from this group [36]. This could be attributed to the presence of synergistic interaction during the period of co-infection [36]. It has been demonstrated earlier that SOCS-1 has a negative regulatory effect on interferon (IFN) mediated JAK-STAT signaling in fish, and that there exists a direct negative interaction between SOCS-1 and STAT-1 and between SOCS-1 and Tyrosine kinase 2 (Tyk2) [28]. SOCS-1 blocks the differentiation of Th1 subset through inhibition of IFN-γ-STAT-1 and IL-12-STAT-4 pathways and hence its deficiency can lead to constitutive expression of IFN-γ and STAT-1 inducing Th1 differentiation, preventing Th17 differentiation [46]. SOCS-1 and SOCS-3 have a negative regulatory role in IFN-γ signaling within human keratinocytes and their overexpression inhibits IFN-γ-induced phosphorylation of IFN-γRα and activation of STAT-1 and STAT-3, leading to impaired IFN-γ-dependent inflammatory responses [47]. The pathogenesis of PKD in rainbow trout is strongly inducing the transcription of Type II IFN-γ [8]. Despite the upregulation of all immune tested genes observed in this study, we found that the relative gene expressions of SOCS-1 and SOCS-3 in the posterior kidney were much higher than JAK-1 and STAT-3. Kumar et al. [42] found that the gene expression of transforming protein RhoA, which regulates the signal transduction pathway of a wide range of cellular processes, was upregulated in the kidney of brown trout infected with *T. bryosalmonae* and suggested that differential modulation of genes may support the parasite development in fish hosts. In our experiment, we studied the induced expressions of the tested JAK-STAT genes in the trout kidneys, during *T. bryosalmonae* infection and found that the gene expressions of SOCS-1 and SOCS-3 were most prominent, which indicated their support for parasite development in the fish host.

It has been reported previously that innate immune response genes namely, IFN-γ, interferon regulatory factor 1 (IRF-1) and inducible nitric oxide synthase (iNOS) in addition to Ubiquitin-like protein 1 were significantly upregulated both in susceptible as well as resistant rainbow trout strains after exposure to *M. cerebralis* [41]. However, STAT-3 and metallothionein B were consistently upregulated in the resistant Hofer strain and remained unchanged in the susceptible TL trout strain following *M. cerebralis* infection [41, 48]. In the present study, we found the expressions of the different immune genes to be significantly lower in the cranial cartilage than in the kidney, a main hematopoietic organ of fish. Therefore, the kidney is characterized as an organ that elicits strong immune response against the invading pathogens. The transcription of SOCS-1 gene was found to be upregulated in the cranial cartilages from 60 dpe to 120 dpe. The highest level of SOCS-1 and SOCS-3 gene expression in the cranium and the highest load of *M. cerebralis* were detected in *Mc*-then-*Tb* co-infected group at 120 dpe, indicating an association between expression of SOCS genes and disease progression and severity. This is in accordance with observations from the first part of this study wherein synergistic effects were elicited during *Mc*-then-*Tb* co-infection along with exacerbated pathological lesions of PKD and WD [36]. The elevated expression of JAK-1 gene in the cranium of single *Mc-*infected and *Mc*-then-*Tb* co-infected group at 60 dpe (Fig. 6), lowered with the course of time. This expression reciprocally correlated with increased expression of SOCS genes in the *Mc*-then-*Tb* co-infected group with the course of time (Fig. 5). Therefore, we suggest that *M. cerebralis* modulates the immune gene expression to overcome the host cellular response. The results obtained from this study will help in understanding the host-pathogen interaction during single and co-infections with PKD and WD.

## Conclusions

In this study, we highlight the SOCS/JAK/STAT signaling pathway and its role in co-infections with two myxozoan parasites. Rainbow trout infected with *M. cerebralis* and then subsequently with *T. bryosalmonae* showed the highest loads of both parasites in the posterior kidneys and cranial cartilages, thereby indicating a synergistic interaction. This study showed differential immunomodulation of SOCS genes and post-receptor JAK/STAT induced genes during myxozoans co-infection when compared to single infection. Our results suggest that *T. bryosalmonae* and *M. cerebralis* alter the JAK/STAT signaling pathway *via* a strong overexpression of SOCS-1 and SOCS-3 genes. However, further studies are required to fully understand the early innate immune response of fish during myxozoans co-infections.

## Additional files

Additional file 1: **Table S1** The correlation of coefficient among different kidney immune genes expression in single and co-infected groups. (DOCX 14 kb)

## Abbreviations

dpe: Days post-exposure
*Mc*: *Myxobolus cerebralis*
PKD: Proliferative kidney disease
RT-qPCR: Reverse transcription quantitative PCR
TAMs: Triactinomyxons
*Tb*: *Tetracapsuloides bryosalmonae*
WD: Whirling disease
