# Supplementary material for: Differential modulation of host immune genes in the kidney and cranium of the rainbow trout (Oncorhynchus mykiss) in response to Tetracapsuloides bryosalmonae and Myxobolus cerebralis co-infections
Source: Parasit Vectors. 2018 May 30;11:326. doi: 10.1186/s13071-018-2912-7 (PMC5977764; doi:10.1186/s13071-018-2912-7)
Supplement: Supplementary file 1 — Table S1 The correlation of coefficient among different kidney immune genes expression in single and co-infected groups. (DOCX 14 kb) [file 13071_2018_2912_MOESM1_ESM.docx]

**Additional file 1** Showing the correlation of coefficient among different kidney immune gene expression in single and co-infected groups.

|  | **SOCS-1/ SOCS-3** | | **SOCS-1/ JAK-1** | | **SOCS-1/ STAT-3** | | **SOCS-3/ JAK-1** | | **SOCS-3/ STAT-3** | | **JAK-1/ STAT-3** | |
| --- | --- | --- | --- | --- | --- | --- | --- | --- | --- | --- | --- | --- |
|  | ***r* value** | ***P* value** | ***r* value** | ***P* value** | ***r* value** | ***P* value** | ***r* value** | ***P* value** | ***r* value** | ***P* value** | ***r* value** | ***P* value** |
| Uninfected control | 0.481 | 0.113 | 0.721 | 0.008^**^ | 0.443 | 0.149 | 0.452 | 0.140 | 0.083 | 0.798 | 0.732 | 0.007^**^ |
| *Tb* single | 0.959 | 0.000^**^ | 0.619 | 0.032^*^ | 0.577 | 0.049^*^ | 0.702 | 0.011 | 0.551 | 0.063 | 0.779 | 0.003^**^ |
| *Mc* single | 0.485 | 0.110 | 0.574 | 0.051 | 0.393 | 0.206 | 0.785 | 0.002^**^ | 0.722 | 0.008^**^ | 0.775 | 0.003^**^ |
| *Tb*-then *Mc* | 0.598 | 0.089 | -0.539 | 0.134 | 0.119 | 0.760 | -0.115 | 0.769 | -0.154 | 0.693 | 0.192 | 0.621 |
| *Mc*-then *Tb* | 0.895 | 0.001^**^ | 0.441 | 0.235 | 0.595 | 0.091 | 0.563 | 0.114 | 0.364 | 0.336 | 0.564 | 0.114 |

* Correlation is significant at *P* < 0.05

** Correlation is significant at *P* < 0.01
